# Control your mind, make affordance available

**DOI:** 10.3389/fpsyg.2015.00096

**Published:** 2015-02-17

**Authors:** Zheng Jin, Yang Lee, Jin Zhu

**Affiliations:** ^1^Institute of Educational Science, Zhengzhou Normal University, Zhengzhou, China; ^2^Department of Psychology, University of California, Davis, CA, USA; ^3^Department of Psychology, Gyeongsang National University, Jinju, South Korea; ^4^Haskins Laboratories, Yale University, New Heaven, CT, USA; ^5^Yunnan University of Nationalities, Kunming, China

**Keywords:** affordance control, ecological approach, action, perception, biofunctional understanding, embodied cognition

## Abstract

Evaluating the affordance–control interpretation of the relationship between performance and object estimation has been proposed by psychophysical and psychonomic studies. This study examined the weight estimation–performance relationship. Individuals with visual impairment or blindness put shots that varied in weight among five scales. In Experiment 1, only the perceived weight was a significant performance constraint. In Experiment 2, the weight was perceived as heavier when the participants’ actions were manipulated through cognitive interpretation. The increase in perceived weight appeared to be related to performance and intrinsically scaled to the action, even when the action was only mental rather than physical. The study’s findings suggest that bodily experience and action are the basis for physical judgments and likely underlie other basic cognitive interpretations of sensory stimuli. This suggestion goes hand in hand with the biofunctional approaches which assume direct experience of the integrated wholeness of one’s body is fundamental for developing other kinds of awareness. Different perspectives from oriental philosophy and psychology are also discussed.

## INTRODUCTION

Action-specific perception theory (or perception–action relation) proposes that people perceive the environment in terms of their ability to act in it (Gibson, 1979; Witt, 2011). It has guided biofunctional studies on the relationship of perception to motor control (e.g., Grezes and Decety, 2001). On the assumption that judgments and productions of target size are good proxies for perceived target size, studies (e.g., Witt et al., 2010) have interpreted the empirically observed performance-estimated size relationship as a performance–perception relationship. In goal-directed tasks, when a player’s performance is better, the player’s estimate of the target size is larger (e.g., Witt and Proffitt, 2005; Witt et al., 2008; Cañal-Bruland and van der Kamp, 2009; Witt and Dorsch, 2009), and the mean estimate is larger for players with more easily performed tasks (e.g., Bhalla and Proffitt, 1999; Proffitt et al., 2003). Accordingly, this thesis has advanced that optical variables, such as those that pertain to hitting a softball or putting a golf ball, are scaled by metrics that emerge from action-specific body organization (e.g., Proffitt and Linkenauger, 2013). Following this argument, a person who performs well perceives targets (e.g., balls, holes) to be larger.

In general, these kinds of experiments share a number of features. First, participants have explicit knowledge of their performance levels prior to their making any size judgments (e.g., Witt and Proffitt, 2005; Witt et al., 2008). Second, the target is of a single size (e.g., a softball or golf cup). Third, participants make size judgments subsequent to extending the full trial complement. Thus, the cognitive reevaluation of the goal size in light of the estimated accuracy, in a sense, is not ruled out, and thus, the link between perceived size and hit rate (i.e., performance) may not be causal. Additionally, it is unknown whether the participants were equally and implicitly aware of both the actual and the retinal sizes of the targets. That is, the dependence of miniature selection on the proximal–distal fit could not be measured. In addition, performance would be expected to improve systematically with target size. In addition to any variations in actual target size, whether there was a performance-estimated size relationship could not be measured. For the above reasons, studies fail to provide a full understanding of the performance-estimated size relationship.

A novel method was thus introduced to provide less-explicit visual knowledge of participants’ performance results (Lee et al., 2012). Participants were confronted with multiple target sizes rather than one, and they estimated target size during each trial rather than after each round. It was found that target size judgments correlate with prior success in hitting the target. In affordances, the manner in which one uses his or her body to interact with the environment affects his or her perception of the environment. A later study supplemented findings by Jin and Lee (2013) and reinforced the notion of affordance-based control. On these grounds, the action-specific approach not only provides robust evidence for the account of affordances but also suggests that perception is mediated by internal processes by demonstrating a new ontology for the behavioral sciences based on biologically relevant affordances rather than physical objects (Jin and Lee, 2013; also see Turvey, 1992; Miller, 2007).

Perception is one’s only information source about the external world and has been explained in many ways (Witt and Sugovic, 2013); larger perceived goal sizes have been associated with performance and are affected by the types of actions. If people are unaware of their performance, then what coordinates the relationship between perception and performance? Further, how can a person reliably produce a beneficial perceptual change (e.g., amplification) in performance? The embodied cognition holds that the nature of the human mind is largely determined by the form of the human body (Shapiro, 2011). Despite the common biofunctional orientation between action-specific perception and embodied cognition, analytic approach that captures the “intuitions” we have about human functioning has not been well developed in these fields. We are invited to more seriously consider the philosophical issue of the “natural kind or proper observable” (Millikan, 1999; Ellis, 2001, also see Rosch, 2000), for which perception is a proxy in these types of experiments. A different paradigm is proposed here of explaining the relationship between action and object estimation, and this explanation might be independent of the physical action status, in contrast to the purely cognitive processes that render affordance available.

## EXPERIMENT 1

### PARTICIPANTS

Nine individuals with blindness (one female and eight males), aged 31–42 years (*M* = 36.88 year, SD = 2.90), were recruited with the assistance of the provincial Disabled Persons Federation. Of the nine participants, six were adventitiously blind and three were congenitally blind. Total blindness was a universal characteristic, and the age at sight loss ranged from 0 (congenitally blind) to 33 years old. The subjects were orally informed of the consent agreement. One participant (female) was excluded from the analysis because she withdrew during the tasks. The participants were paid 50 Chinese yuan (CNY) as compensation. Because the sample size was similar to the study by Lee and colleagues (Lee et al., 2012; Jin and Lee, 2013) and smaller than the study by Witt and colleagues (Witt and Proffitt, 2005; Witt et al., 2008), for a direct comparison, we report rank-order correlation coefficients (see *results* below).

### MATERIALS

**FIGURE 1:**
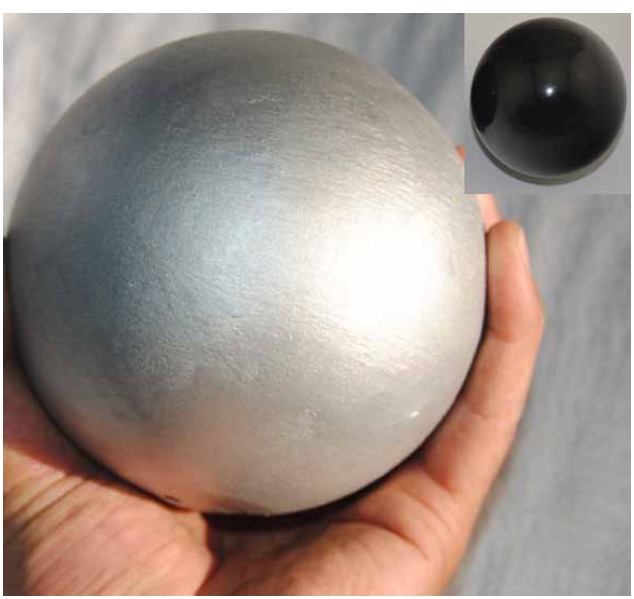
**A shot and a solid ball used in Experiment 1**.

The experiment was conducted in a field with ample surroundings. The participants were instructed to put five artificial shots with varying weights as far as they could. The standard weight is 3 kg (3000 g); the weights of the five shots used in this study were 1000, 2000, 3000, 4000, and 5000 g (Figure 1). The diagonal lines of these shots were identical (13 cm). To provide their weight estimates, the participants selected one of eleven solid balls (diagonal lines, 13 cm), which varied from 500 to 5500 g in 500 g increments.

### PROCEDURE

**FIGURE 2:**
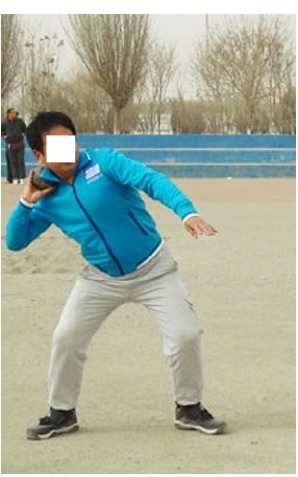
**Putting the shot without run-up**.

When the task began, participants were required to hold the shots for approximately 10 s and then use a standard putting method without run-up (Figure 2), as taught by the experimenters, before proceeding to the experiment. Immediately after putting, the participants were required to use the same hand they had used to put to quickly weigh eleven solid balls in turn and then provide an approximate estimate of the weight of the shot they had just put. The instruction “estimate a weight that feels most like the shot you just put” was given. The perceived weight measurements presumed that the participant was implicitly aware of both the physical (the shots) and proximal (the solid balls) weights at equal levels (Carlson, 1960). The separate reports of the two weights are provided independently (Ashby and Townsend, 1986; for a discussion of decisional separability and signal detection, see Oberle and Amazeen, 2003).

There were 25 putting trials for each participant, five trials for each shot. The solid balls were randomly assigned, and they varied from trial to trial. The participants were not provided the actual weights of the shots, and the shots’ weights also varied randomly over the trials. After each trial, the shot was taken away, and a new shot was provided by the experimenters. The putting distance was recorded as the performance level. The participants could choose to pause briefly between trials to avoid fatigue.

We used three experimenters: one assisted and instructed the participants throughout the procedure, one recorded the participants’ weight estimations, and one measured the putting distance near the “target” (beyond).

### RESULT AND DISCUSSION

The separate reports between the weights of solid ball and physical shots can be provided independently. However, the *R square* value between the perceived and proximal weights of 1000, 2000, 3000, 4000, and 5000 g were 0.69 for the eight participants, *t*(38) = 9.20, *p* = 3.34e-11 (*r*_s_ = 0.83, *N* = 40, 1-tailed *p* = 1.13e-11), which reveals that perceived weight depended on the actual shot weight, as well as implying that the participants systematically related their weighing of the solid balls based on the fit between the shot and solid ball weights.

**FIGURE 3:**
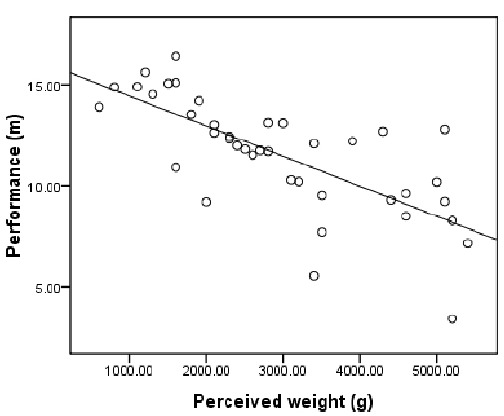
**Mean putting distance for the eight participants against perceived weight (i.e., implicit judgments of proximal weight, but c.f. Lee et al., 2012)**.

The participants judged the shots as heavier when the actual shots were heavier. The putting distance was also greater when the shots were lighter, *r*^2^ = 0.30, *t*(38) = –4.05, *p* = 2.43e-4 (*r*_s_ = –0.54, *N* = 40, 1-tailed *p* = 1.66e-4). Importantly, the putting distance correlated with perceived shot weight (Figure 3). However, if the perceived weight is considered to be an independent variable rather than dependent variable, multiple regression analysis revealed that only the perceived weight predicted the performance, *r*^2^ = 0.53, β = –0.02, *p* = 1.24e-7, and the actual shot weight was not a significant constraint for the putting distance, *p* = 0.404.

The performance–perception relationship could be interpreted using the empirically observed performance–estimation relationship. Many studies have scaled performance using metrics from action-specific body organization (e.g., Witt et al., 2010). Previous studies also confirm that unitary coordination, which comprises bodily status and action processes (e.g., muscle work), is responsible for the performance–estimation relationship. This coordination determines the performance accuracy (level) relative to the target. The results are in agreement with those provided by Lee et al. (2012). For example, a person standing by a creek may consider it too wide to jump across, but a person who is running and approaching the creek may have a high chance of a successful jump because the creek is visually narrower when a person is running than when he is standing, which “affords” jumping. As perception improves, the person begins to clearly feel how the body is connected and how actions are naturally integrated systems rather than merely coordinated and controlled separate parts. In this study, putting form is primarily registered by the haptical system (Turvey and Carello, 2011). This suggests that perception was scaled based on the perceived levels of coordination and control for any given action.

Our mental busyness, however, is a distinct distraction from accurate perception and awareness. In fact, it appears as though all of our senses are dulled by thinking (Brown and Cordon, 2009). Therefore, it might be that participants’ cognitive form during putting constrains their immediately subsequent proximal weight judgments. Accordingly, a manipulation that mentally affects the haptically perceptible form, rather than the form *per se*, should also affect perceived weight. The second experiment aimed to examine this hypothesis.

## EXPERIMENT 2

### PARTICIPANTS AND MATERIALS

Ten blind men (one female, nine males; *M* age = 38.30 year, SD = 3.4) from the same population participated in Experiment 2. Seven were adventitiously blind, and three were congenitally blind. Total blindness was a universal characteristic, with age at loss of sight ranging from 0 (congenitally blind) to 31 years. The same *apparatus* from Experiment 1 was used.

### PROCEDURE

Participants were told that if they found the task to be too emotionally taxing or uncomfortable, they could terminate it at any time. The same experimental procedure from Experiment 1 was used with one exception: Experiment 2 included an additional condition, action *imagination*. The participants were not informed that they would perform the task in two ways. One task comprised half of the trials; Experiment 2 replicated the detailed procedure from Experiment 1.

The other task constituted the remainder of the trials. The participants were ostensibly asked to take a hearing test, and they were invited to wear a pair of special (Bluetooth) earplugs and listen to an audio document. The participants also received instruction through the earplugs. Prior to the task, the participants were told to imagine that they were walking a long distance for exercise; simultaneously, an audio recording was played with the continuous sound of a blind-guiding stick rhythmically tapping on a road surface, the sound of breathing, a tread sound, and an indication voice stating “you have walked X meters” that was repeated at 18-s intervals using an audio-play device with a remote wireless control. At one minute and a half (00:01:30), an indication voice with the message “you are going to walk uphill on a slope” was presented. As the recording advanced, the stick-tapping rhythm became slower, more gasping accompanied a slower tread sound, and the “X” communicated by the indication voice became shorter. When the rhythm, gasp, and tread frequency reached a certain level, the audio recording did not vary (00:02:30) but played continuously. The participants were then instructed to proceed through the task. The audio recording was constantly played until the task ended. The indication voice stating “you have walked X meters on the slope” and “you are going to finish the exercise in about X minutes” kept repeating, replacing the previous voice, at a certain interval. All of the indication voice messages were triggered by an experimenter. All of the “X” and “certain intervals” in the indication voice were controlled by the experimenter depending on specific conditions. In all of the *imagination* trials, the volume was adjusted to suit the participants. For half of the participants, the task involved an *imagination* preceded by the other task. In this experiment, each of the five shots (1, 2, 3, 4, and 5 kg) for one task consisted of only two trials. The relevant correlation results involving performance (i.e., putting distance) are discussed in Experiment 1; we did not measure performance in Experiment 2.

Participants received a simple questionnaire that asked about the effectiveness of the imagination task. We asked whether their imagination had involved more events than exercise and whether it had involved more actions than walking. No participant reported more events that were not related to exercise, and three participants reported more actions, i.e., trotting and running. After each task was completed, we also assessed the participants’ moods—“How do you feel right now?”— on a scale of 1 (very good) to 9 (very bad).

### RESULT AND DISCUSSION

There were no effects on the mood test, for *imagination* task (*M* = 3.70, SD = 1.64), and for *normal* task (*M* = 4.30, SD = 0.95). The main effects of *imagination* (two tasks), task order and actual shot weight (five weights) as well as the interaction effects were tested using the analysis of variance (ANOVA). The mean perceived weights were 2990.2 g (SD = 1250.65) for the normal task and 3220.60 g (SD = 1300.97) for the *imagination* task. The difference was significant, *F*(1,8) = 30.76, *p* = 0.001, η^2^ = 0.79. A strong significant effect from the actual shot weight was also observed, *F* (4,32) = 203.37, *p* = 0.2.83e-22, η^2^ = 0.96. *Post hoc* multiple comparisons using the Bonferroni correction showed significant differences (*p*s < 0.01) between all shot weights: 1 kg (*M* = 1440.5 g; SE = 110.92), 2 kg (*M* = 2140.00 g; SE = 120.04), 3 kg (*M* = 3220.00 g; SE = 140.65), 4 kg (*M* = 3890.50 g; SE = 100.10), and 5 kg (*M* = 4840.50 g; SE = 70.98). The analysis did not reveal any other significant effects. Table 1 shows the perceived weight for each actual shot from the different tasks.

**Table 1:** **Mean estimation of proximal weight (SD) as a function of physical weight and task type in Experiment 2**.

	Proximal weight (g)	
Physical weight (kg)	With imagination	Without imagination
1	1510 (420.80)	1380 (390.94)
2	2270 (400.57)	2010 (380.72)
3	3280 (460.62)	3160 (460.24)
4	4100 (410.37)	3690 (350.73)
5	4970 (290.08)	4720 (300.84)

In Experiment 2, the participants exhibited significantly increased weight perceptions when they imagined voluntary actions accompanied by the audio aids. We did not physically manipulate the haptically perceived levels in Experiment 2; however, the perceived levels that were cognitively invited concurrently increased the proximal weight judgments. The results from Experiment 2 provide evidence for the speculation that in a given trial, the weights judged in Experiment 1 were scaled to the coordination and control action levels for that trial; the levels were cognitively rather than physically manipulated. Perception is cognition because people’s actions (e.g., running, walking, etc.) occur in their minds and in reality. Humans can use this cognition as a basis for adapting to (or better accomplishing) a task in a given environment. The concept of this ability has a clear position in oriental philosophy (see *general discussion* below).

## GENERAL DISCUSSION

Based on studies by Fajen (2007), Fajen et al. (2009), Lee et al. (2012), and Jin and Lee (2013), putting is an example of affordance-based control. For blind persons, the shots’ weights varied with the forms of touching, holding, weighing, and putting. The perceived weights reported by the participants were implicit reports of their “*puttability*” estimates. Shockley et al. (2004) suggested that physical dimension estimates are elliptical indications of affordances. In this study, the weights varied systematically with the masses distributed to the participants’ hands; thus, manipulation affected the hand-wielded objects’ perceived *movability* or *controllability*. Affordances might be sufficiently fundamental that they necessarily constrain weight judgments (Fajen and Phillips, 2012). Thus, heavier shots are more difficult to put than lighter shots, and accordingly, perceiving a shot’s put-to-be reflects the perception of that shot’s weight.

Under the traditional paradigm, affordance is nested in environment. The following question ensues: what could make the outer affordance available? A person with visual impairment may depend more on a (haptical or olfactory) perceptual system for survival than a normal individual. These individuals perceive objects not only through bodily actions but also by controlling affordance **before** the action. Improving the ability to feel and be mindful of actions can be fundamental to how one improves performance. The process is grounded in awareness: the essential tool of the mind that firmly roots it back into the body. Not only does this awareness foster a body that is free from restriction, but it also serves as a tool for calming the overactive mind. During the entire putting process, a blind man would exhibit a *puttable* effect, which may support the general thesis that affordance has an effect. As we have reiterated, if perception is cognition because people form perceptions in their minds, in reality, manipulations that affect the cognitive form should also affect weight judgments. Fatigue and muscle strength are measures of overall bodily energy, which can be considered a latent variable. One might describe the effects of neurotransmitters on this latent “bodily energy,” in contrast to focusing on molecular or cellular actions. Research shows that people’s physiological and psychosocial resources influence their visual perception (e.g., Harber et al., 2011). This approach is similar to studies on the higher-level cognitive-emotional-physical effects of drugs such as antidepressants, the physiological actions of which are not well-understood.

In this study, the participants were activated by an action in which the putters’ perceived levels of coordination and control were presumably lower. In the mind, this action may have constrained the proximal weight estimates; if so, the possibility that many other manipulations, such as emotional, motivational, and procedural activation, among others, affect perception cannot be ruled out. For example, in a controversial study, participants for whom an elderly stereotype was primed walked more slowly than did control participants (Bargh et al., 1996). Our results do not robustly contribute to the argument about priming effect replicability (Doyen et al., 2012; Yong, 2012). Paraphrasing *imagination* in the present study, manipulations that only affect perceived levels of coordination and control also affect the judgments of proximal targets, goals, or objects, which provides organisms with affordances that suggest that the proximal weights judged were intrinsically scaled to the current perceptual systems (e.g., haptic and auditory). This suggestion is somewhat consistent with the study by Lee and Schnall (2014), although it demonstrates that power, which drives potential strategies for gaining resources, can affect perception; people see the physical world as reflecting the difficulties posed by their lack of potential actions.

Many people experience the relationship between judging the “actable-ness” of a target and performance when the form is correct and they perform well. However, in empirical studies, differences or changes in perception observed under conditions without external behavior may be considered behavioral information and may be used to assess participants’ effects on behavior. Similar to the aforementioned blind person’s special survival skills, normal people who are well-trained are more apt to perceive the actable-ness of an object for better performance (e.g., Lee et al., 2012; Experiment 1) because they can adjust their perceptual systems to control the affordances and obtain high levels of perception. Deliberately sensing the body brings people directly into contact with the present moment, which trains the mind to focus as if on automatic pilot. Modern neuroscience literature suggests that this trained “presence” actually builds new neural pathways within the brain and is akin to the highly prized “zone” (Robinson, 2004) that top athletes enter when they are in good form. The findings in this study also share some similarities with various “mindfulness” practices that are fashionable at the moment. People can manipulate perceived performance levels in a given environment, and this ability might render outer affordance available.

Behavior is traditionally considered a means to control the environment, but this view ignores the fact that an object can be perceived through action. People sit after they confirm the distance between a table and a bench; people may also first sit down and then reevaluate or adjust their distance. Although the initial stimulus is always perception, the interaction continues back and forth between perception and action. Biological studies have found that self-generated sensations are attenuated by a predictive mechanism and suggest that the nervous system’s ability to predict an action’s sensory consequences may be used for other mechanisms in addition to its role in sensory attenuation (e.g., Bays et al., 2006). Our ability to mentally rehearse movements before performing them may be attributable to this type of prediction. Cognition guides behaviors that acquire the perceptions necessary for new behaviors. The two behaviors before and after the cognitive process (e.g., reappraisal) communicate environmental information. Gibson (1979) conceptualized this information as *survival-related symbols* given by the environment to an organism. Post-Gibson researchers have also advocated the notion that affordances are dispositional *symbols* of physical objects that necessarily actualize related actions under appropriate circumstances (e.g., Caiani, 2014). The concept of survival is similar to that of *Chi* energy (are also known as *Kih* paradigm) in oriental philosophy (Lee et al., 2007; Jin and Lee, 2013), which defines affordance-control ability. Since ecological account showed *direct perception* and denied the need for any internal processing of biologically relevant cues to perceive meaningful affordances, it seemed to offer nothing to the analytically oriented cognitive science (e.g., Ullman, 1981). Coordination and control are not only external actions but also level variables of internal affordance-control ability.

It may be difficult to explore to a larger extent what demands might be required for more successful performances. In this study, people may have perceived that the shots were heavier if their *Chi* had been energetic. Action is not only external but also internal; it even exists in a mental form, the mental processes for which may be regarded as a level of the *Chi* variable. *Chi* paradigm is more like a general theoretical framework that claims a bodily experience; action is the basis for making physical judgments and likely underlies all other basic cognitive interpretations of sensory stimuli. This goes hand in hand with the biofunctional understanding which assume direct experience of the integrated wholeness of one’s body is fundamental for developing other kinds of awareness (e.g., Rosch, 1999). Specifically, the basis for perceptions may be understood in biofunctionalism which approaches the body as a dynamical system seen in their functional, rather than anatomical capacity, even at the sublevel (e.g., a bodily organ such like brain, Iran-Nejad, 2000). It appears that we cannot help separating mind from body in Western philosophical dogma. In order to understand the integrated nature of the mind and body, we must recognize that the mind, as a function of the brain, is essentially embodied. If we approach the mind and body as an integrated unit, we cannot separate training our muscles from our mental activity because they are unavoidably linked. Training the body perceptively necessitates quietening and training the mind as it actively engages in its observant and embodied nature. *Chi* paradigm offers a potential analytic approach to capture the intuition of wholeness in terms of biofunctionalism within the cognitive sciences.

### Conflict of Interest Statement

The authors declare that the research was conducted in the absence of any commercial or financial relationships that could be construed as a potential conflict of interest.
